# Geraniol, a natural monoterpene, ameliorates hyperglycemia by attenuating the key enzymes of carbohydrate metabolism in streptozotocin-induced diabetic rats

**DOI:** 10.1080/13880209.2017.1301494

**Published:** 2017-03-22

**Authors:** Sukumar Babukumar, Veerasamy Vinothkumar, Chandrasekaran Sankaranarayanan, Subramani Srinivasan

**Affiliations:** Department of Biochemistry and Biotechnology, Faculty of Science, Annamalai University, Annamalainagar, India

**Keywords:** Diabetes mellitus, glucose 6-phosphatase, fructose 1, 6-bisphoshpatase, hexokinase

## Abstract

**Context:** Geraniol, an acyclic monoterpene alcohol is found in medicinal plants, is used traditionally for several medical purposes including diabetes.

**Objectives:** The present study evaluates the antihyperglycemic potential of geraniol on key enzymes of carbohydrate metabolism in streptozotocin (STZ)-induced diabetic rats.

**Materials and methods:** Diabetes was induced in experimental rats, by a single intraperitoneal (i.p) injection of STZ [40 mg/kg body weight (b.w.)]. Different doses of geraniol (100, 200 and 400 mg/kg b.w.) and glyclazide (5 mg/kg b.w.) were administrated orally to diabetic rats for 45 days. Body weight, food intake, plasma glucose, insulin, blood haemoglobin (Hb), glycosylated haemoglobin (HbA_1c_), hepatic glucose metabolic enzymes and glycogen were examined.

**Results:** The LD_50_ value of geraniol is 3600 mg/kg b.w. at oral administration in rats. Administration of geraniol in a dose-dependent manner (100, 200, 400 mg/kg b.w.) and glyclazide (5 mg/kg b.w.) for 45 days significantly improved the levels of insulin, Hb and decreased plasma glucose, HbA_1C_ in diabetic-treated rats. Geraniol at its effective dose (200 mg/kg b.w.) ameliorated the altered activities of carbohydrate metabolic enzymes near normal effects compared with two other doses (100 and 400 mg/kg b.w.). Geraniol treatment to diabetic rats improved hepatic glycogen content suggesting its anti-hyperglycemic potential. Geraniol supplement was found to preserve the normal histological appearance of hepatic cells and pancreatic β-cells in diabetic rats.

**Discussion and conclusions:** The present findings suggest that geraniol can potentially ameliorate key enzymes of glucose metabolism in experimental diabetes even though clinical studies used to evaluate this possibility are warranted.

## Introduction

Diabetes mellitus (DM) is a metabolic disorder characterized by hyperglycaemia with disturbances in carbohydrate, fat and protein metabolism, secondary to an absolute or relative lack of insulin (Fatima et al. 2010). It is a common public health problem throughout the world, with an estimated prevalence of 383 million people in 2013, expected to increase to 552 million people by 2030 (Colosia et al. 2013). Particularly, in India the number of diabetic people is expected to raise from 62 million to 101.2 million by 2030 (Kaveeshwar & Cornwall 2014). Several studies have demonstrated that alterations in carbohydrate metabolism play a vital role in the pathogenesis of diabetes and accelerate the development of diabetic complications (Soumya & Srilatha 2011).

Liver is an insulin-dependent tissue, which plays a pivotal role in blood glucose homeostasis. The key enzymes of glycolysis and gluconeogenesis are disturbed, leading to the chronic hyperglycaemia (Berg et al. 2001). Hyperglycaemia characterized by an abnormal postprandial blood glucose level has been linked to the onset of type 2 DM, associated with oxidative dysfunction and failure of various organs, especially the eyes, kidneys and nerves (Prathapan et al. 2012). STZ is a selective pancreatic β-cell genotoxicant used to induce experimental diabetes in model organisms. STZ-induced diabetic animals exhibit many of the complications similar to that of human diabetes (Srinivasan & Ramarao 2007). Therefore, the beneficial effects of antidiabetic principles are well-studied in these animal models.

Recently, undesirable side effects have been reported from the pharmacological drugs used for the treatment of diabetes. The search for antidiabetic drugs, focusing on plant-based medicine gains importance due to their potential benefits and provides ways for the treatment of human diabetes to reduce undesirable side effects. Plants provide a predominant resource for a huge number of conventional medicines that have been in existence for hundreds of years in countries like India (Kondeti et al. 2010). Terpenes are a group of secondary plant metabolites that are widespread in nature and have significant hypoglycaemic effect, which is well documented in several experimental studies. Geraniol possesses pharmacological activities such as antimicrobial (Lorenzi et al. 2009), antioxidant (Tiwari & Kakkar 2009), antilipid peroxidative (Chen & Viljoen 2010), anticancer (Pattanayak et al. 2009) and anti-inflammatory properties (Ji et al. 2002). The objective of the present study is to evaluate the antihyperglycemic efficacy of geraniol by assessing the activities of key metabolic enzymes of glucose metabolism in STZ-induced diabetic rats.

## Materials and methods

### Drugs and chemicals

Geraniol (98%) and STZ were obtained from Sigma Chemical Company (St. Louis, MO). All additional chemicals utilized were obtained either from Hi Media (Mumbai) and SD-Fine Chemicals (Mumbai). All chemicals used were of analytical grade.

### Animals and diet

Adult male albino Wistar rats with body weight ranging from 180 to 200 g were procured from Central Animal House, Department of Experimental Medicine, Rajah Muthiah Medical College and Hospital, Annamalai University, Annamalainagar, Tamil Nadu, India, and were acclimatized for two weeks prior to start the experiments. They were maintained in a controlled environment under standard conditions of temperature and humidity with light–dark cycle (12 h light/dark cycle). Throughout the experimental period, the animals were fed with a balanced commercial pellet diet (Hindustan Lever Ltd., Mumbai, India) and water *ad libitum.* The experimental protocol was approved by the Institutional Animal Ethical Committee of Annamalai University (Reg. No.987/2013).

### Experimental induction of diabetes

Experimental diabetes was induced in overnight fasted rats by single intraperitoneal injection (i.p.) of STZ (40 mg/kg b.w.) dissolved in 0.1 M of citrate buffer (pH 4.5) Rakieten et al. (1963). As STZ is capable of inducing fatal hypoglycemia due to massive pancreatic insulin release, rats were provided with 10% glucose solution after STZ administration for the next 24 h to overcome drug-induced hypoglycaemia. Rats with blood glucose level ≥250 mg/dL were considered diabetic and were used in the study.

### Experimental design

After acclimatization for a period of one week, the rats were randomly divided into seven groups of six animals in each group. Geraniol was dissolved in corn oil (2.5 mL/kg b.w.) and an aqueous solution of glyclazide (5 mg/kg b.w.) was administered orally once in a day for 45 days.

Group 1: Normal (vehicle treated)

Group 2: Normal + geraniol (400 mg/kg b.w.)

Group 3: Diabetic control (vehicle treated)

Group 4: Diabetic + geraniol (100 mg/kg b.w.)

Group 5: Diabetic + geraniol (200 mg/kg b.w.)

Group 6: Diabetic + geraniol (400 mg/kg b.w.)

Group 7: Diabetic + glyclazide (5 mg/kg b.w.)

### Sample collection

The initial and final body weights of the various groups were recorded. At the end of the experimental period, the animals were fasted overnight, sacrificed by cervical decapitation. Blood samples were collected in tubes containing potassium oxalate and sodium fluoride (3:1) mixture for the estimation of plasma glucose, insulin, haemoglobin (Hb) and glycosylated haemoglobin (HbA_1C_) levels. Liver and kidney tissues were excised immediately and washed in ice-cold saline, homogenized in Tris-HCl buffer (0.1 M, pH 7.5), centrifuged (3000 rpm) and the supernatant was collected for various biochemical estimations. A portion of the tissues was used for histopathology studies. The pancreas was dissected out, used for histological and immunohistochemical studies.

### Assessment of oral glucose tolerance

On the day prior to sacrifice, oral glucose tolerance test (OGTT) was performed in all the groups. Blood samples were obtained from the lateral tail vein of overnight fasted experimental animals. Successive blood samples were taken at 0, 30, 60, 90 and 120 min after glucose administration of 2 mg/kg b.w. of glucose solution (Young et al. 1995).

### Biochemical assays

Plasma glucose levels were estimated using a commercial kit (Sigma Diagnostics Private Limited, Baroda, India) by the method of Trinder (1969). Hb and HbA_1c_ were estimated using diagnostic kit (Agappe Diagnostic Private Limited, Kochi, India) (Bisse & Abraham 1985).

### Estimation of carbohydrate metabolic enzymes

Hexokinase was assayed by the method of Brandstrup et al. (1957). Glucose 6-phosphate dehydrogenase was assayed by the method of Ellis and Kirkman (1961). Glucose 6-phosphatase was assayed by the method of Koide and Oda (1959). Fructose 1,6-bisphosphatase activity was measured by the method of Gancedo and Gancedo (1971).

### Histopathological study

For the histological study, the pancreas was excised immediately perfused with cold physiological saline and fixed in 10% formalin. Then, dehydrated on treatment with a series of different concentration of ethanol and embedded in paraffin wax. Sections of 3–5 μm thickness were cut using a microtome and stained with haematoxylin and eosin stain. The specimens were evaluated with a light microscope. All histopathological changes were examined by eminent pathologist.

### Immunohistochemistry

Immunohistochemical staining was done to confirm the presence of insulin positive cells in the islets of pancreas in all rats, as described by Ozougwu et al. (2013). Paraffin sections were serially cut into 5 μm thickness and placed on microscopic slides sealed with poly-l-lysine (Sigma, USA). The tissue was fixed on the microscopic slide in a 37 °C oven for 1 h. Then, the tissues were deparaffinized by rinsing in xylene for three times and dehydrated with two rinses in absolute alcohol and two rinses in 95% ethanol for 3 min each before being washed with phosphate buffer saline (PBS) and distilled water for 5 min each. The tissue sections were then incubated for 15 min in 3% H_2_O_2_ in methanol to quench the endogenous peroxidase. The sections were then washed in PBS for 5 min and excess PBS was wiped around the tissues. The sections were blocked by incubating with diluted normal serum for 20 min and excess serum was blotted from the sections. For the detection of insulin, sections were incubated with primary antibody (Rabbit polyclonal anti-insulin antibody from Cell Signaling, Danvers, MA) diluted to 1:100 in PBS for 60 min, and sections were washed with PBS for 5 min. Excess PBS was wiped from the slides. Then, the sections were processed by indirect immunoperoxidase technique using a One-Step Polymer HRP Detection kit (Biogenex, The Hague, The Netherlands) with secondary antibodies using haematoxylin as the counterstain. Evaluation of immunohistochemical staining was made by the examination of 10 islets for all groups of rats using a Confocal Scanning Microscope (Carl Zeiss, Oberkochen, Germany).

### Statistical analysis

The experimental results were expressed as means ± SD and subjected to one-way analysis of variance (ANOVA), using a computer software package (SPSS version 11.5; SPSS Inc., Chicago, IL) and the comparisons of significant differences among the groups were performed using Duncan’s post has multiple range test (DMRT), *p* < 0.05 was considered as significantly different between means.

## Results

### Effect of geraniol on changes in body weight, food and fluid intake

Table 1 shows the changes in the levels of body weight of control and experimental groups. At the end of the experimental period, the body weight was significantly decreased in diabetic rats when compared with normal control rats. Food and fluid intake were significantly elevated in diabetic control rats throughout the experimental period. On the other hand, diabetic rats treated with geraniol (100, 200 and 400 mg/kg b.w.) showed significantly decreased food and fluid intake with improved body weight to near normal which is comparable with glyclazide group.

**Table 1.** Effect of geraniol on body weight, fluid and food intake of normal control and experimental rats.

	Body weight (g)		Fluid intake (mL/rat/day)		Food intake (g/rat/day)	
Groups	Initial	Final	Before	After	Before	After
Normal control	206.34 ± 15.71	214.30 ± 16.32^a^	67.52 ± 5.14	67.23 ± 5.12^a^	15.68 ± 1.19	18.09 ± 1.38^a^
Normal + geraniol (400 mg/kg b.w.)	207.47 ± 15.89	215.84 ± 16.53^a^	69.09 ± 5.29	71.54 ± 5.48^a^	16.15 ± 1.24	19.28 ± 1.48^a^
Diabetic control	211.13 ± 16.08	136.60 ± 10.40^b^	76.44 ± 5.82	187.91 ± 14.31^b^	56.88 ± 4.33	72.28 ± 5.50^b^
Diabetic + geraniol (100 mg/kg b.w.)	210.75 ± 16.14	188.89 ± 14.4^c^	73.27 ± 5.61	126.53 ± 9.69^c^	53.12 ± 4.07	38.90 ± 2.98^c^
Diabetic + geraniol (200 mg/kg b.w.)	208.28 ± 15.95	197.14 ± 15.5^d^	74.99 ± 5.74	97.63 ± 7.48^d^	55.76 ± 4.27	30.26 ± 2.09^d^
Diabetic + geraniol (400 mg/kg b.w.)	207.99 ± 15.93	198.25 ± 15.72^d^	70.88 ± 5.43	94.42 ± 7.23^d^	54.97 ± 4.21	29.12 ± 2.00^d^
Diabetic + glyclazide (5 mg/kg b.w.)	206.33 ± 15.80	200.43 ± 15.89^d^	74.0 ± 5.67	90.96 ± 6.97^d^	57.14 ± 4.38	25.23 ± 1.93^d^

Values are given as mean ± SD from six rats in each group.

Values not sharing a common superscript letter^(a–d)^ differ significantly at *p* < 0.05 (DMRT).

### Effect of geraniol on OGTT, plasma glucose and insulin

**Figure 1.**
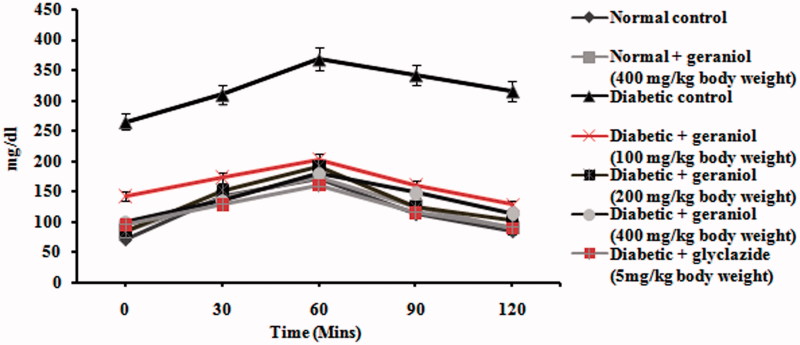
Effect of geraniol on the levels of oral glucose tolerance test in normal control and experimental rats.

OGTT conducted on control and experimental rats are shown in Figure 1. OGTT of diabetic rats showed a highly pronounced elevation of glucose at fasting state and at 30, 60, 90 and 120 min after oral glucose intake as compared with control rats. Administration of geraniol showed a significant decrease in blood glucose levels, which signifies insulin secretion.

**Figure 2.**
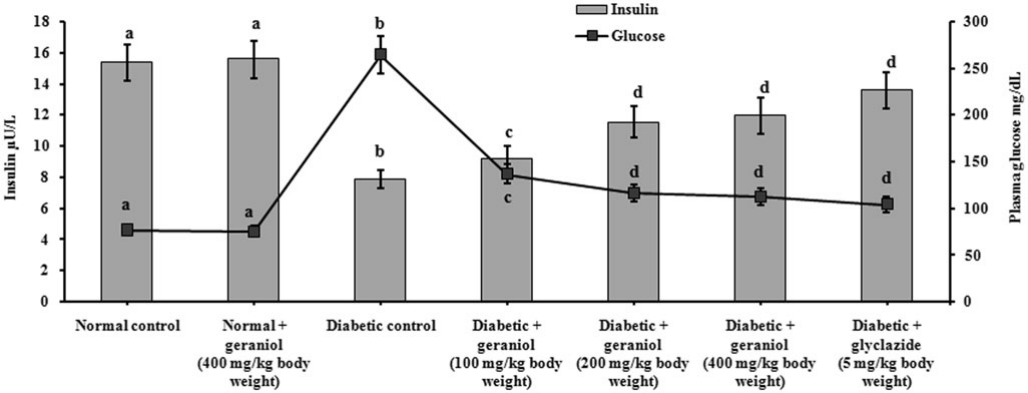
Effect of geraniol on the levels of plasma insulin and glucose in normal control and experimental rats.

The effect of geraniol on the levels of plasma glucose and insulin in experimental rats are showed in Figure 2. Glucose levels were elevated and plasma insulin was significantly decreased in diabetic rats when compared with normal control rats. Oral administration of geraniol (100, 200 and 400 mg/kg b.w.) for 45 days significantly decreased blood glucose and increased plasma insulin to near normal, as compared with glyclazide treated rats. Geraniol at a dose of 200 and 400 mg/kg b.w. showed a highly significant effect than the lower dose (100 mg/kg b.w.). Based on these data, the effective dose was fixed as 200 mg/kg b.w. and further studies were carried out at this dose.

### Effect of geraniol on levels of Hb and HbA_1C_

Table 2 shows the levels of Hb and HbA_1C_ in normal control and diabetic rats. Decrease in Hb level and an increase in HbA_1C_ level were observed in diabetic rats. These values were reverted back towards near normal on treatment with geraniol and glyclazide.

**Table 2.** Effects of geraniol on the levels of Hb and HbA_1C_ in normal control and experimental rats.

Parameters/Groups	Hb (g/dL)	HbA_1c_ (%Hb)
Normal control	15.32 ± 1.32^a^	6.46 ± 0.45^a^
Normal + geraniol (200 mg/kg b.w.)	15.00 ± 1.32^a^	6.15 ± 0.44^a^
Diabetic control	7.9 ± 0.55^b^	15.42 ± 0.88^b^
Diabetic + geraniol (200 mg/kg b.w.)	12.01 ± 0.88^c^	11.22 ± 0.88^c^
Diabetic + glyclazide (5 mg/kg b.w.)	12.95 ± 0.88^c^	12.03 ± 0.88^c^

Values are given as mean ± SD from six rats in each group.

Values not sharing a common superscript letter^(a–c)^ differ significantly at *p <* 0.05 (DMRT).

### Effect of geraniol on the activities of carbohydrate metabolizing enzymes

Table 3 shows the effects of geraniol on the activities of glycolytic and gluconeogenic enzymes in the liver and kidney tissues of normal control and experimental rats. Significantly elevated activities of gluconeogenic enzymes such as glucose 6-phosphatase and fructose 1, 6-bisphosphatase as well as decreased activities of hexokinase and glucose 6-phosphate dehydrogenase were found in liver tissue of diabetic rats when compared with normal control rats. Oral administration of geraniol (200 mg/kg b.w.) as well as glyclazide (5 mg/kg b.w.) to diabetic rats reversed the activities of these enzymes to near normal.

**Table 3.** Effect of geraniol on the activities of carbohydrate metabolic enzymes in the tissues of normal control and experimental rats.

Parameters/groups	Normal control	Normal + geraniol (200 mg/kg b.w.)	Diabetic control	Diabetic + geraniol (200 mg/kg b.w.)	Diabetic + Glycazide (5 mg/kg b.w.)
Glycolytic enzyme					
Hexokinase					
Liver (units/g protein)	155.61 ± 11.92^a^	154.23 ± 11.75^a^	112.16 ± 8.59^b^	138.60 ± 10.56^c^	140.31 ± 10.75^c^
Glucose 6-phosphate dehydrogenase					
Liver (×10^−4^ ml U/mg protein)	5.71 ± 0.43^a^	5.30 ± 0.41^a^	3.15 ± 0.24^b^	4.75 ± 0.36^c^	4.62 ± 0.35^c^
Kidney (μg/mg protein)	4.55 ± 0.35^a^	4.60 ± 0.35^a^	2.95 ± 0.22^b^	3.82 ± 0.29^c^	3.90 ± 0.30^c^
Gluconeogenic enzyme					
Glucose 6-phosphatase					
Liver (μg/mg protein)	0.20 ± 0.02^a^	0.16 ± 0.01^a^	0.45 ± 0.03^b^	0.29 ± 0.02^c^	0.26 ± 0.02^c^
Kidney (μg/mg protein)	0.19 ± 0.01^a^	0.17 ± 0.01^a^	0.49 ± 0.04^b^	0.25 ± 0.02^c^	0.27 ± 0.02^c^
Fructose 1,6-bisphosphatase					
Liver (μg/mg protein)	0.31 ± 0.02^a^	0.35 ± 0.03^a^	0.67 ± 0.05^b^	0.43 ± 0.03^c^	0.47 ± 0.04^c^
Kidney (μg/mg protein)	0.28 ± 0.02^a^	0.31 ± 0.02^a^	0.55 ± 0.04^b^	0.47 ± 0.04^c^	0.43 ± 0.03^c^

Values are given as mean ± S.D from six rats in each group.

Values not sharing a common superscript letter^(a–c)^ differ significantly at *p <* 0.05 (DMRT).

### Effect of geraniol on histology of pancreas

**Figure 3.**
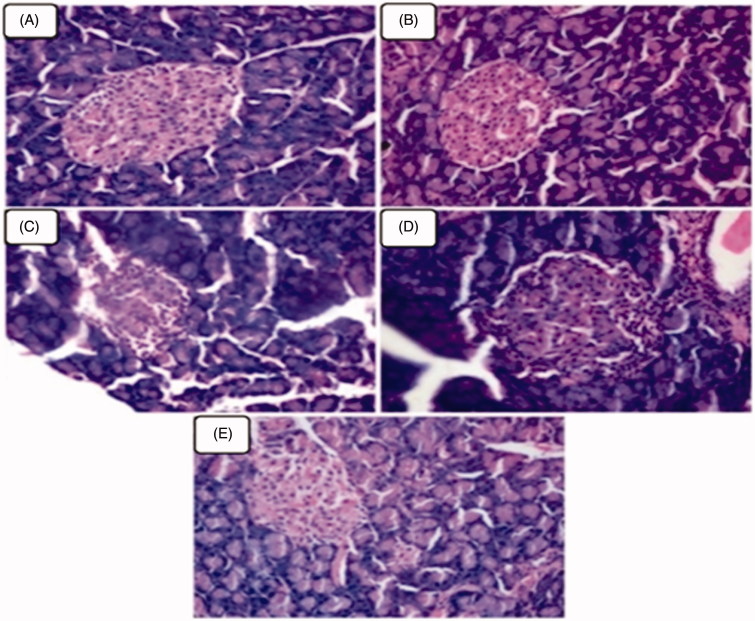
(A–E) Represents the microphotographs of pancreatic tissues of normal control and experimental rats in each group (haematoxylin and eosin staining, 40×). (A and B) Normal control and normal control + geraniol (200 mg/kg body weight) treated rats showed normal appearing pancreatic exocrine glands and ducts with Islet of Langerhans. (C) Diabetic rats showing fatty infiltration and shrinkage of islet cells. (D and E) Pancreatic tissues from diabetic + geraniol (200 mg/kg b.w.) and diabetic + glyclazide (5 mg/kg b.w.) treated rats shows normal appearing pancreatic acini with absence of dilation and prominent hyperplastic of islets.

Histopathological studies on the pancreatic islets of experimental rats are shown in Figure 3(A–E). Diabetic rats (Figure 3(C)) showed fatty infiltration with shrinkage of islet cells. Oral administration of geraniol (Figure 3(D)) and glyclazide (Figure 3(E)) restored pancreatic acini with the absence of dilatation.

### Effect of geraniol on immunohistology of pancreas

**Figure 4.**
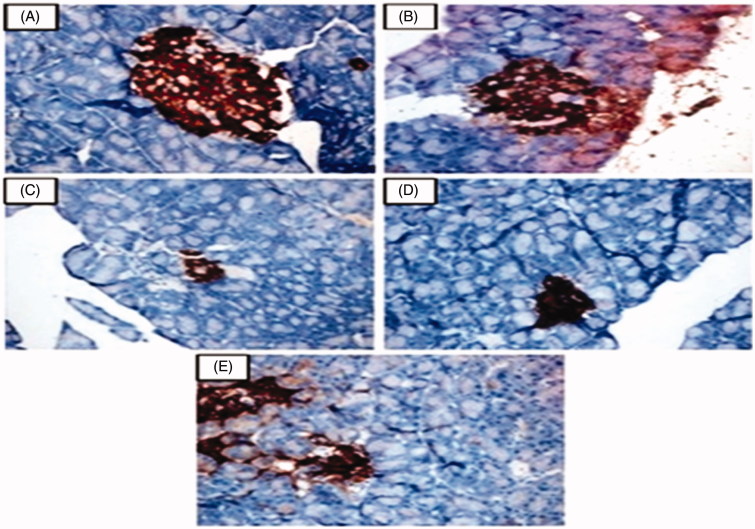
This figure shows the immunoexpression pattern of insulin secreting cells in pancreatic islets observed in the normal control and experimental rats in each group (40×). (A and B) Normal control and normal control + geraniol (200 mg/kg b.w.) treated rats showed normal immunohistochemical expression of insulin secreting cells in pancreatic islets. (C) Diabetic control showed decreased staining of insulin secreting cells in pancreatic islets. (D and E) Diabetic + geraniol (200 mg/kg b.w.) and diabetic + glyclazide (5 mg/kg b.w.) treated rats showed increased insulin secreting cells in pancreatic islets.

A decrease in the number of insulin-immunoreactive β-cells was observed in the diabetic control group. Immunohistochemical section of the pancreatic islets of diabetic rats treated with geraniol (200 mg/kg b.w.) and glyclazide (5 mg/kg b.w.) showed increased insulin-immunoreactive β-cells (Figure 4(D,E)). This clearly suggests that after treatment with geraniol, positive immunoreactions of β-cells for anti-insulin antibodies were obviously increased in numbers with deep brown granules as interpreted in Figure 4(D).

Table 4 depicts the % of insulin positive staining areas in pancreatic β-cells of normal, control and experimental rats in each group. Normal control and geraniol control rats revealed no significant difference in the % area of positive stain while a significant decline was observed in diabetic rats. The % area of immunoreactivity of insulin was substantially increased in geraniol- (200 mg/kg b.w.) and glyclazide-treated (5 mg/kg b.w.) diabetic rats. This indicates the potential insulinotropic effect of geraniol.

**Table 4.** Effects of geraniol on the levels of immunoreactive cells of insulin in pancreatic islets of normal and experimental rats.

Parameters/groups	% of Insulin positive cells
Normal control	66.14 ± 4.29^a^
Normal + geraniol (200 mg/kg b.w.)	69.23 ± 4.84^a^
Diabetic control	39.97 ± 3.74^b^
Diabetic + geraniol (200 mg/kg b.w.)	67.20 ± 5.04^c^
Diabetic + glyclazide (5 mg/kg b.w.)	70.06 ± 5.60^c^

Values are given as mean ± S.D from six rats in each group.

Values not sharing a common superscript letter^(a–c)^ differ significantly at *p* < 0.05 (DMRT).

## Discussion

Type 2 DM is one of the most common chronic and progressive metabolic disorders with impaired insulin secretion, insulin resistance with augmented hepatic glucose production (Jainandunsing et al. 2015). This study evaluates the effect of geraniol by measuring the activities of key enzymes involved in carbohydrate metabolism in the liver and kidney of control and experimental rats. Recently several studies reported the biological activities and important applications of geraniol (Choi et al. 2000; Ji et al. 2002; Duncan et al. 2004; Vinothkumar & Manoharan 2011). Pancreas is the primary organ involved in sensing the organism’s diet and energetic states via glucose concentration in the blood and in response to elevated blood glucose, releases insulin to control glycaemic status (Sundaram et al. 2013). STZ is a nitrosurea derivative, preferentially taken up by pancreatic β-cells via low affinity GLUT-2 transporter and causes DNA alkylation followed by the activation of poly ADP ribosylation leading to depletion of cytosol concentrations of NAD^+^ and ATP resulting in destruction of β-cells of pancreas. An insufficient release of insulin leads to hyperglycemia and results in diabetic complications (Grover et al. 2002).

STZ-induced diabetic rats showed increase in water and food intake with a significant loss of body weight. In this study, diabetic rats showed marked reduction in their body weight when compared to normal rats, which could be due to the unavailability of carbohydrate as energy source (Sundaram et al. 2014). We also observed an increase in food and water intake in diabetic rats that result from a chronic reduction in glucose utilization by the cells and considerable loss of glucose through urine. Oral administration of geraniol significantly improved glycaemic control which prevented the loss of body weight and improved food and fluid intake in diabetic control rats in a dose-dependent manner.

An OGTT is a sensitive measure of early abnormality in glucose regulation than HbA_1c_ or plasma glucose_._ An increase in the plasma glucose level after glucose load during the OGTT was observed in diabetic rats. The data obtained from the OGTT clearly show that the blood glucose level remains high even after 120 min in diabetic control rats (Ceriello 2005). However, in geraniol-treated diabetic rats the blood glucose levels reached a peak and returned again to fasting levels after 120 min. This revealed that the increased glucose tolerance in geraniol-treated STZ rats is due to insulin secretion from existing β-cells and augmented glucose transport and utilization.

Blood glucose control is an important component in delaying or preventing acute or long-term diabetic complications (Gandhi & Sasikumar 2012). In the present study, we observed a low level of plasma insulin in STZ-induced rats denoting perturbations in β-cells function, whereas geraniol-treated rats showed increased plasma insulin levels, indicating that geraniol treatment improved β-cell function in STZ rats. The increased insulin levels after the treatment of geraniol in diabetic rat may be related to the closure of K^+^-ATP channels and stimulation of Ca^2+^ influx, an initial key step in insulin secretion from remnant β-cells. These results are in harmony with Lone and Yun (2016), who reported that, a monoterpene, limonene reduced glucose levels and increased insulin secretion in β-cells of diabetic rats.

HbA_1C_ is considered as a gold standard marker for accurate and reliable measurement of fasting glucose, which is strongly associated with the prognosis of the diabetes (Lakshmi et al. 2009). The decreased level of Hb in diabetic rats is mainly due to the increased formation of HbA_1C_. In DM, the excess blood glucose reacts with Hb to form HbA_1C_. The increase in HbA_1C_ is directly proportional to the blood glucose level (Nain et al. 2012). Profound studies well documented that during diabetes, increased glucose and dicarbonyl compounds react with haemoglobin to form advanced glycation end products, which leads to the development of diabetic complications (Bunn et al. 1978; Turk 2010). Treatment with geraniol and glyclazide to diabetic rats significantly improved the levels of Hb and decreased HbA_1C_ when compared to normal control rats. This is due to the restoration of blood glucose with a reduction in the glycosylation of Hb.

Glucose homeostasis is related to endogenous production and utilization of glucose by target tissues (Wilding 2014). Insulin regulates the activities of various enzymes such as hexokinase, glucose-6-phosphate dehydrogenase, glucose-6-phosphatase and fructose 1,6-bisphosphatase which are involved in carbohydrate metabolism (Prasath & Subramanian 2014). Hexokinase is one of the key enzymes, in glycolysis, which catalyzes the conversion of glucose to glucose 6‐phosphate in liver and plays a pivotal role in the maintenance of blood glucose homeostasis (Matschinsky 2009). Being an insulin‐dependent enzyme, the hepatic hexokinase activity of diabetic rats is almost entirely inhibited or inactivated due to the lack of insulin (Selvaraja & William Ndyeabura 2011). This impairment results in a marked reduction in the rate of glucose oxidation via glycolysis in tissues with decreased glucose removal from the blood, thus ultimately leads to hyperglycaemia. Several studies documented that hexokinase activity is significantly decreased in STZ-induced diabetic rats (Malini et al. 2011; Ramachandran & Saravanan 2013; Vanitha et al. 2014). Oral administration of geraniol to STZ‐induced diabetic rats resulted in a significant reversal in the activity of hexokinase, thereby increases the oxidation of glucose. Profound studies reported that hexokinase is the potential target for new treatment strategies for the management of type 2 diabetes (Thiebaud et al. 1982; Murali & Saravanan 2012).

Glucose 6-phosphate dehydrogenase is the primary rate limiting enzyme of hexose mono-phosphate shunt and produces NADPH required for the maintenance of glutathione (Kondeti et al. 2010). It has an important role in pancreatic β-cell function and its survival (Cernea & Dobreanu 2013). Previous studies demonstrated that the lack of insulin results in the reduction of glucose 6-phosphate dehydrogenase activity in STZ-induced diabetic models (Baltrusch et al. 2012). Our results corroborate with these findings. In DM, decreased activity of glucose 6-phosphate dehydrogenase affects the concentration of NADPH and induces oxidative stress in various tissues (Stanton 2012). In our study, administration of geraniol and glyclazide significantly improved the activity of glucose 6-phosphate dehydrogenase in liver and kidney tissues of diabetic rats. It provides hydrogen, which binds NADP^+^ and produces NADPH and enhances the synthesis of fats from carbohydrate, which finally decreases the plasma glucose level.

During starvation, the activities of chief regulatory enzymes of gluconeogenesis, glucose-6-phosphatase and fructose 1, 6-bisphosphatase in liver and kidney were increased. These enzymes play a vital role in the regulation of blood glucose levels. In diabetic state, enhanced activities of gluconeogenic enzymes increases hepatic endogenous glucose production, which leads to chronic hyperglycemia (Nordlie et al. 1999). In the current study, diabetic rats showed a significant increase in the activities of gluconeogenic enzymes. Oral administration of geraniol to diabetic rats significantly reversed the activities of gluconeogenic enzymes. Our results are concomitant with Deepa and Anuradha (2011) who reported that limonene a monoterpene reduced the activities of gluconeogenic enzymes in STZ-induced diabetic rats.

The morphology and number of insulin-positive β-cells in the islets were studied using the immunohistochemical method. The results of the present study showed marked reduction in insulin-positive β-cells in the pancreatic tissue of diabetic rats compared to normal rats. The percentage of insulin positive cells per islet was significantly increased in geraniol- and glyclazide-treated diabetic rats as compared with diabetic control rats. This observation indicates that geraniol and glyclazide increases the number of insulin positive cells in the pancreas of diabetic-treated rats. From the histopathological studies, the changes observed in the pancreas of diabetic rats were significantly reversed on treatment with geraniol and glyclazide. Our results are in agreement with Rathinam et al. (2014) who also observed that, a monoterpene, myrtenal shows similar results in histological changes of pancreas. Thus, the present study demonstrated the antidiabetic potential of geraniol in STZ-induced type-2 diabetic rats.

## Conclusions

We concluded that geraniol ameliorates carbohydrate metabolism and restores glucose homeostasis by altering the activities of enzymes involved in glucose production and utilization. It also protects pancreatic β-cells and exhibits significant insulinotrophic activity in experimental diabetic rats. In addition, immunohistochemical staining of pancreas confirms insulin secretion from remnant pancreatic β-cells in geraniol-treated diabetic rats. This suggests that geraniol exerts significant antidiabetic properties; however, further studies are essential to understand the plausible molecular mechanism of geraniol in diabetic rats.
